# State of Art and Perspective of Calcium Phosphate-Based Coatings Coupled with Bioactive Compounds for Orthopedic Applications

**DOI:** 10.3390/nano15151199

**Published:** 2025-08-05

**Authors:** Matteo Montesissa, Viviana Tommasini, Katia Rubini, Marco Boi, Nicola Baldini, Elisa Boanini

**Affiliations:** 1 Biomedical Science and Technologies and NanoBiotechnologies Lab, IRCCS Istituto Ortopedico Rizzoli, 40136 Bologna, Italy; 2 Department of Chemistry “Giacomo Ciamician”, University of Bologna, 40129 Bologna, Italy; 3 Department of Biomedical and Neuromotor Sciences, University of Bologna, 40126 Bologna, Italy

**Keywords:** coating, deposition techniques, orthopedic surgery, antibacterial effect, antitumoral effect, pro-osseointegration effect

## Abstract

The aim of this review is to investigate the possibility of fabricating coatings functionalized with bioactive molecules. These coatings are interesting when applied to biomedical devices, particularly in the orthopedic field. In fact, the application of calcium phosphate-based coatings on the surface of implanted devices is an effective strategy to increase their osteoinductive and osseointegrative properties. Several coating fabrication technologies are presented, including chemical deposition and physical methods. The application of bioactive molecules in combination with calcium phosphate coatings may improve their osteointegrative, antibacterial, and antitumor properties, therefore increasing the performance of implantable devices.

## 1. Introduction

The improvement of biomedical devices represents a crucial element to increase their performance and impact on patient daily life. In particular, regarding the orthopedic and dentistry fields, the use and research on calcium phosphate-based materials is widely diffuse due to their similarity with the mineral component of bone tissue, mainly composed of carbonated hydroxyapatite [1,2,3]. These properties and similarity can be exploited to manufacture and apply coatings following the biomimetism principle on metal implants and prosthesis surfaces, principally composed of titanium alloys and chromo-cobalt-molybdenum alloys [4,5]. In fact, the application of calcium phosphate-based coatings can increase the primary stability of the implant with a firm bonding with the surrounding host bone tissue [1]. This behavior is important to increase the osteointegration properties and avoid aseptic loosening and fibrous tissue formation between the implant surface and the surrounding bone tissue, eventually leading to implant failure [6]. However, the possibility to use different calcium phosphates in coating fabrication is important to explore their properties in terms of crystallinity, solubility, and ion release in the peri-implant environment [7,8].

Furthermore, the combination of the calcium phosphate materials with specific bioactive compounds and molecules can improve the performance once applied in the human body [9]. In particular, the use of antibiotics can favor the antibacterial properties of the implant against several bacterial strains involved in peri-implant infections, such as *Staphylococcus aureus* and *Staphylococcus epidermidis*. Alternatively, several bioactive compounds and drugs can be applied to improve the treatment of some diseases, such as osteoporosis and cancers.

Here, we explore different aspects related to improving metallic materials surfaces used in the orthopedic field to better understand the current state and future perspectives in the use of calcium phosphate coatings coupled with different bioactive compounds and molecules.

## 2. Calcium Phosphates Materials

Among the most extensively studied and employed materials for bone tissue replacement or regeneration are calcium phosphates (CaPs), a family of minerals comprising calcium cations (Ca^2+^) in combination with orthophosphates (PO_4_^3−^, HPO_4_^2−^, H_2_PO_4_^−^). These compounds represent the predominant inorganic component of human bone (~60 wt%) and dental enamel (up to 90 wt%). Their key properties include excellent biocompatibility, attributable to their compositional similarity to human bone, as well as osteoconductivity and osteointegration, which significantly influence cell adhesion and proliferation. Furthermore, in contrast to bioinert materials, calcium phosphates can establish a direct chemical bond with bone, a process initiated by the adsorption of proteins onto their surface, which in turn mediate cell growth and promote biochemically driven osteogenesis [10,11,12]. The primary mechanism underlying their bioactivity lies in the partial dissolution of the material and the consequent release of calcium and phosphate ions. This process increases the local ionic concentration and promotes the precipitation of a new layer of biological apatite on the surface of the biomaterial, which serves as a bridge between the implant and the host tissue. However, their intrinsic brittleness and poor mechanical strength, resulting from the ionic nature of their bonds, represent a significant limitation. This aspect is particularly critical in high-load applications, where considerable impact resistance is required. For this reason, CaPs are mainly used as coatings or as fillers for bone defects in non-load-bearing areas [10,13]. In Table 1, all the CaPs are reported with their principal characteristics.

### 2.1. Dicalcium Phosphates (DCPA, DCPD)

Dicalcium phosphates have recently raised the attention due to their biocompatibility, biodegradability, and osteoconductivity. Two crystalline forms exist: DCPD (also known as Brushite) and its anhydrous form DCPA (also known as monetite). DCPA is less soluble than DCPD and can be crystallized from aqueous solutions at 100 °C or after heat treatment of the hydrated form DCPD [14,22]. DCPD can be easily prepared at physiological temperature and can be converted into octacalcium phosphate (pH ≈ 6–7), or poorly crystalline hydroxyapatite (pH > 7) after hydrolysis reaction [14,22].

### 2.2. Tricalcium Phosphates (TCP)

α-Tricalcium phosphate (α-TCP) represents one of the most interesting forms for biomedical applications, particularly in bone regeneration. Chemically, the term TCP (tricalcium phosphate) is used in its strictest sense to refer to phases with a composition of Ca_3_(PO_4_)_2_, characterized by a Ca/P molar ratio of 1.5. α-TCP is distinguished as the high-temperature phase of TCP. It is typically obtained through the calcination of β-TCP at temperatures above 1125 °C, followed by rapid cooling to prevent the reverse transformation into the more stable β-phase at room temperature. Despite sharing the same chemical composition, α-TCP and β-TCP exhibit significant differences in crystal structure and, most notably, solubility. Under physiological conditions, α-TCP can react and transform into hydroxyapatite, thus serving as a guide for bone tissue growth [17,23,24]. β-TCP exhibits a more compact and symmetric crystal structure compared to α-TCP, resulting in a higher theoretical density of approximately 3.067 g/cm^3^, whereas α-TCP has a lower theoretical density of around 2.814 g/cm^3^ (Table 1). This difference is primarily due to the more open of α-TCP, which forms at high temperatures (above ~1125 °C) and is metastable at room temperature.

β-tricalcium phosphate (β-TCP) is one of the most extensively studied and utilized phases in the field of biomaterials for bone regeneration, owing to its osteoconductive and, in some cases, osteoinductive properties. β-TCP exhibits an intermediate solubility between that of hydroxyapatite and α-TCP, making it particularly suitable for applications requiring gradual resorption over time. A distinctive feature of β-TCP is that it cannot be directly precipitated from aqueous solutions due to its chemical and physical stability conditions. However, it can be prepared via thermal decomposition of carbonated apatite (CDHA) or through solid-state reactions between acidic calcium phosphates, such as DCPA or DCPD, and CaO or CaCO_3_ at temperatures exceeding 800 °C [18,25]. Biologically, β-TCP has demonstrated a favorable behavior toward bone cells: it promotes osteoblast proliferation more effectively than metallic substrates. This activity is attributed to its lower interfacial energy compared to hydroxyapatite, a characteristic that enables β-TCP to induce the precipitation of an apatite-like layer when incubated in ionic aqueous solutions, thus simulating a physiological microenvironment. This apatite-like layer acts as a chemical bridge between the implant and the surrounding bone tissue, promoting integration and tissue regeneration [26].

### 2.3. Amorphous Calcium Phosphate (ACP)

Amorphous calcium phosphate (ACP) constitutes a distinct class within the family of calcium phosphate compounds, characterized by the absence of long-range crystalline order and exhibiting glass-like physical properties. In contrast to crystalline phases such as hydroxyapatite (HA) or tricalcium phosphate (TCP), ACP possesses variable chemical compositions but share a high reactivity, which makes it particularly attractive for biomedical applications. Depending on the synthesis conditions, ACP can be categorized based on the temperature at which it is produced. Low-temperature ACP, typically obtained by precipitation from aqueous solutions, often serves as a transient, unstable precursor during the formation of more stable crystalline phases such as HA. High-temperature ACP, though less common in biological environments, is generated through more complex processes and is investigated for specific technological applications. In physiological conditions, ACP plays an important role in tissue mineralization processes. In particular, ion-substituted forms containing Na^+^, Mg^2+^, carbonate, or pyrophosphate have been detected in pathological calcifications of soft tissues. The rapid release of these ions can stimulate early stages of bone formation and promote the precipitation of biological apatite, thus acting as a trigger for cellular differentiation. However, the high solubility that underpins their biological activity also presents certain limitations. A sudden release of ions may cause transient fluctuations of local pH, creating acidic or basic microenvironments that can impair cell adhesion and proliferation in the short term, and compromise cell viability in the long term if not properly controlled. Consequently, ACP is often employed as a temporary precursor or incorporated into hybrid coatings alongside more stable phases, to modulate biological responses in a more predictable and controlled manner [13].

### 2.4. Tetracalcium Phosphate (TTCP)

Tetracalcium phosphate (TTCP, Ca_4_(PO_4_)_2_O) is the most basic calcium phosphate phase commonly studied and applied in biomedical field. Despite its high basicity, TTCP exhibits greater solubility in aqueous environments compared to HA, making it suitable for applications requiring a faster, yet controlled, ionic release. Unlike phases such as CDHA or ACP, TTCP cannot be synthesized through aqueous precipitation and must instead be produced via solid-state reactions at elevated temperatures, typically above 1300 °C. In the context of orthopedic implant coatings, TTCP is often considered an undesirable secondary phase, particularly in plasma-sprayed HA coatings, as its presence can adversely affect the chemical stability and biological performance of the material. Therefore, precise optimization of deposition parameters is crucial to minimize the formation of TTCP and other secondary phases [20,21].

### 2.5. Octacalcium Phosphate (OCP)

OCP plays an important role in the in vivo formation of apatitic biominerals and is employed both for bone defect implantation and as a coating material [27,28]. OCP is frequently observed as a transient or intermediate phase during the precipitation of thermodynamically more stable calcium phosphates, such as hydroxyapatite (HA) or calcium-deficient hydroxyapatite (CDHA), from aqueous solutions. From a crystallographic perspective, OCP exhibits a triclinic structure composed of apatitic layers, characterized by calcium and orthophosphate ion arrangements similar to those in HA, alternated with hydrated layers structurally analogous to those found in DCPD [29]. OCP shows a relatively higher solubility compared to other calcium phosphates, which explain its hydrolysis reaction to hydroxyapatite when incubated in aqueous solution [30]. Biologically, OCP has been identified as a stable component of human dental and urinary calculi. Although OCP has not been directly observed in vascular calcifications, it has been strongly suggested as a precursor phase to the biological apatite found in natural and prosthetic heart valves [20]. When used as a coating, OCP enables the formation of homogeneous surface layers with variable thicknesses, adapting to substrate geometry and promoting osteointegration through its gradual transformation into apatite under biological conditions [27].

### 2.6. Hydroxyapatite (HA)

Hydroxyapatite (HA) is one of the most extensively investigated and utilized calcium phosphate phases for biomedical applications, owing to its excellent biocompatibility, bioactivity, and osteoconductivity. Its stoichiometric formula is Ca_5_(PO_4_)_3_(OH), although it is commonly expressed as Ca_10_(PO_4_)_6_(OH)_2_ to reflect the composition of its hexagonal unit cell. Thermodynamically, HA is the second most stable and least soluble calcium phosphate, following fluorapatite (Fap). Despite its relatively low solubility, HA provides abundant nucleation sites for new apatite crystal formation in calcium and phosphate rich environments or directly within biological fluids, enhancing integration with bone tissue [13]. Synthetic HA is recognized as a bioactive material capable of establishing a strong chemical bond with host bone, resulting in early implant stabilization and superior fixation to surrounding tissues. Nevertheless, HA-based ceramics are inherently brittle and exhibit poor mechanical performance, particularly in aqueous environments. As a result, bulk HA is unsuitable for load-bearing orthopedic applications [31]. Mechanical strength has been shown to increase with the Ca/P atomic ratio, reaching a maximum at approximately 1.67, corresponding to stoichiometric HA, and decreasing sharply when the ratio exceeds this value. Strength also decreases exponentially with increasing porosity; however, tailoring the pore geometry can partially mitigate this effect. It is noteworthy that porous HA exhibits significantly lower fatigue resistance compared to its dense counterpart [32]. To address these limitations, HA is frequently applied as a coating on metallic or polymeric implant substrates. This strategy combines the excellent bioactivity of HA with the superior mechanical properties of the underlying substrate, thereby enhancing the overall performance of the implant [33,34]. In addition to being bioactive, HA is osteoconductive, non-toxic, non-immunogenic, and crystallographically similar to natural bone mineral, particularly when incorporating appropriate levels of carbonate substitution [35]. However, in the absence of specific stimuli, HA is generally not osteoinductive. Its biological properties can be tuned via ionic substitutions. For instance, replacing phosphate groups with carbonate increases solubility and bioactivity, while substituting hydroxide groups with fluoride enhances chemical stability but reduces resorption rates. Cationic substitutions, such as the incorporation of magnesium in place of calcium, may also exert favorable biological effects by supplying trace ions essential for cellular functions [36].

### 2.7. Carbonated Deficient Hydroxyapatite (CDHA)

Nanocrystalline biomimetic apatite is receiving increasing attention. Carbonated deficient hydroxyapatite (CDHA), sometimes also referred to as poorly crystalline hydroxyapatite (PHA), represents a low-crystallinity, non-stoichiometric form of hydroxyapatite, whose composition closely resembles that of biological apatite found in human bone tissue. Owing to these features, CDHA is considered highly promising for bone regeneration and artificial bone substitute applications. It can be easily synthesized through low-temperature precipitation by simultaneously adding calcium- and phosphate-containing solutions into boiling water, followed by prolonged boiling of the suspension. During this process, initially precipitated amorphous calcium phosphate (ACP) restructures and transforms into CDHA. An alternative route to precipitation is the hydrolysis of α-tricalcium phosphate (α-TCP), which allows the production of CDHA with controlled properties [19].

The thermal behavior of CDHA is strongly dependent on the molar Ca/P ratio. Upon heating above 700 °C, different phase transformations are observed: a CDHA with Ca/P = 1.5 converts entirely into β-tricalcium phosphate (β-TCP), whereas CDHA with a Ca/P ratio between 1.5 and 1.67 transforms into a biphasic mixture of hydroxyapatite (HA) and β-TCP, commonly known as biphasic calcium phosphate (BCP). It is important to note that non-substituted, pure CDHA does not naturally occur in biological systems. Instead, in physiological environments, it always exists in an ionically substituted form, incorporating ions such as Na^+^, K^+^, Mg^2+^, and Sr^2+^ (in place of Ca^2+^), CO_3_^2−^ (in place of PO_4_^3−^), or F^−^, Cl^−^, and CO_3_^2−^ (in place of OH^−^) [37]. This ionic substitution, together with the presence of structural water, leads to the formation of so-called biogenic apatite, which plays a critical role in bone remodeling and mineralization processes [20,38]. Biogenic apatite, therefore, significantly differs from synthetic stoichiometric HA (Ca/P ratio of 1.67) in terms of chemical composition, stoichiometry, crystal size and morphology, degree of crystallinity, degradation rate, and overall biological performance. It is a carbonated, non-stoichiometric, calcium-deficient material with reduced crystallinity. In particular, biogenic apatite crystals are smaller and possess a higher specific surface area. Researchers have also developed alternative methods to obtain biogenic apatite at low cost from natural and sustainable sources, such as mammalian bones or fish bones [39].

## 3. Calcium Phosphates Functionalization

Calcium phosphates (CaPs) are widely recognized as reference biomaterials in orthopedic and maxillofacial applications due to their biocompatibility and osteoconductive properties. The functionalization of CaPs with specific ions, molecules, and biomolecules enhances cell interactions to promote osteogenesis and angiogenesis, counteracts the onset of infections, and reduces tumor recurrence. Functionalization can be achieved through three main strategies: ionic substitution, doping, and chemical bonding (i.e., adsorption, grafting):

- Ionic substitution involves the replacement of ions (Ca^2+^, PO_4_^3−^, and OH^−^) within the HA crystal lattice with other elements and/or molecules;
- Doping refers to the controlled introduction of impurities into the interstitial sites or dislocations of the crystal lattice;
- Chemical bonding (including van der Waals, covalent, and ionic interactions) enables surface modification of HA through the anchoring of functional molecules via specific interactions.

The most extensively studied functionalization approach by chemists has been the substitution of various mono- and divalent metal cations (e.g., Na^+^, Ag^+^, Cu^2+^, Sr^2+^, Mg^2+^) at the Ca^2+^ site within the HA crystal lattice [40]. Anion substitution (e.g., carbonate, silicate [41], fluoride, selenite can occur at the hydroxyl site (type-A substitution), the phosphate site (type-B substitution), or simultaneously at both sites (type-AB substitution) [42].

Carboxylate-containing molecules (such as citrate, hydroxycitrate, and glutarate) can be adsorbed onto the HA surface [43]. The results showed that hydroxycitrate strongly inhibits HA crystallization more effectively than citrate, leading to the formation of smaller nanoparticles with lower crystalline order. In contrast, glutarate exhibited a weaker inhibitory effect on HA growth, promoting the formation of initial plate-like crystals of octacalcium phosphate (OCP), which subsequently matured into nanorods.

Drugs such as bisphosphonates (e.g., alendronate [44,45], zoledronate [46], risedronate [47]) can interact with the Ca^2+^ ions in HA through bidentate chelation involving deprotonated oxygen atoms of the bisphosphonate anion, without significantly altering the HA crystal structure. Alendronate has also been grafted onto octacalcium phosphate [48]. The same authors synthesized Sr^2+^-substituted HA conjugated with zoledronate, enabling the simultaneous presence of two inhibitors of bone resorption [49]. In this study, it was observed that a single-phase HA could be obtained with Sr concentrations in solution up to 20% as a function of zoledronate concentration.

Flavonoids such as quercetin (3,3′,4′,5,7–pentaidrossi-flavone) include anti-oxidant and antinflammatory response [50,51], as well as capability to prevent bone loss [52,53]. Hydroxyapatite (HA) was functionalized with quercetin using two distinct approaches [54]: (i) direct synthesis, which resulted in the formation of nanocrystals containing up to 3.1 wt% quercetin, albeit with partial oxidation of the flavonoid and a consequent reduction in its antioxidant activity; and (ii) phase transformation from monetite, enabling incorporation of up to 1.3 wt% quercetin with minimal structural alterations and preservation of significant antioxidant activity. Both materials were evaluated using an advanced in vitro model simulating the bone microenvironment (including osteoblasts, osteoclasts, and endothelial cells). The composites promoted osteoblast proliferation and differentiation, inhibited osteoclast formation (osteoclastogenesis), and supported endothelial cell growth and differentiation (angiogenesis).

Peptides such as arginine-glycine-aspartic acid (RGD), which is one of the most common peptide motifs responsible for cell adhesion to the extracellular matrix (ECM), have been anchored onto the HA surface through both physical and chemical methods, utilizing free –OH sites [55]. Regarding physical adsorption, the HA surface was immersed overnight in an aqueous RGD solution (100 µg/mL), allowing peptide adsorption via weak (non-covalent) interactions. Subsequently, the samples were sonicated in ultrapure water to remove loosely bound RGD peptides. For the chemical approach, the HA surface was covalently grafted using a silanizing agent. Specifically, HA was first treated with ethanol and then immersed in a solution of (3-aminopropyl) triethoxysilane (APTES, silanizing agent) for 8 h under agitation. APTES introduced reactive amine groups onto the surface, enabling the subsequent stable covalent bonding of the RGD peptide [56]. However, the use of an RGD motif has two main issues: (i) mimetic peptides may act as partial agonists as well as competitive antagonists of integrins, displaying, in vivo, interactions or competition with endogenous processes [57]; and (ii) the RGD motif can bind to multiple integrin classes, so that the specificity and selectivity of cell activation could be highly limited [58]. New β-lactam-based molecules able to modulate cell adhesion on targeting different integrins [59,60]. The structure of the new ligands was designed with the β-lactam ring as a conformational restriction motif that could gain a favorable alignment on the receptor and molecularly suited for integrin affinity and selectivity. Strontium-substituted hydroxyapatite (SrHA) was functionalized with two monocyclic β-lactams (compounds 1 and 2), selected for their activity toward specific integrins [61]. The loading of β-lactams onto SrHA was modulated by varying the polarity of the loading solution, resulting in a content ranging from 3.5% to 24% by weight for compound 1 and from 3.2% to 8.4% for compound 2. The new composite materials were fully characterized, and their release profiles in water were investigated, revealing an initial rapid release followed by a sustained release phase.

## 4. Calcium Phosphates Coating Techniques

Nanostructured calcium phosphate (CaP) coatings are promising due to their submicrometric thickness, which helps to avoid mechanical discrepancies that lead to fracture. Additionally, nanostructure promotes host cell adhesion and proliferation, and may even offer cues for faster osteogenic differentiation [12,62,63].

It is well-established that coatings for orthopedic implants should possess low porosity, strong cohesive strength, good substrate adhesion, high crystallinity, and excellent chemical and phase stability. The properties of CaP can yield coatings with varying bioactivity and longevity. Currently, there is broad consensus that the chemical purity of HA should be as high as possible (≥95%), with a Ca/P ratio of 1.67 [64]. The ability of a CaP coating to adsorb proteins and facilitate cell adhesion is influenced by its surface roughness (<100 nm), crystallinity, solubility, phase content, particle size, surface charge, and surface energy. Studies have shown that more crystalline surfaces promote osteoblast attachment and proliferation [65], while more soluble surfaces release ions more rapidly, promoting bone growth [66]. However, high solubility and dissolution rates can negatively affect the long-term reliability of the coating on an implant [67,68].

Osteointegration also depends on the chemistry and physical characteristics of the CaP surface. The topographical features of Ca/P coatings, such as the size and shape of granules, affect the differentiation of osteogenic cells, leading to high alkaline phosphatase (ALP) and osteocalcin synthesis activity [69]. Another key property is the resorption rate of CaP in the body, which depends on its porosity and crystallinity. Porous materials accelerate dissolution and can be replaced by new bone within a period ranging from 3 to 36 months. Porosity is also crucial for bone tissue growth as it allows for cell colonization [70]. Additionally, the wettability (hydrophilicity) of CaP is essential for regulating osteogenesis, as it modulates cell activity and guides cells toward hydrophilic surfaces [65,71].

The specifications for CaP coatings influence the resulting mechanical properties of the implant, such as cohesive and bonding strength, tensile strength, shear strength, Young’s modulus, residual stress, and fatigue life. Modifications to these variables can yield coatings with variable bioactivity and longevity [13,69].

Since the early 1980s, scientific research has focused intensively on the modification of bone implant surfaces through the application of inorganic calcium/phosphorus (Ca/P)-based coatings [72]. In this framework, numerous deposition approaches have been developed, which can be broadly classified into two main categories: wet chemical techniques and plasma-based physical techniques.

Wet techniques include methods such as sol-gel processing, electrophoresis, and biomimetic precipitation from simulated body fluids (SBF) or other calcifying solutions. These processes rely on liquid solutions containing—among others—calcium and phosphate ions, which are subsequently converted into solid films through chemical reactions or thermal treatments. Such approaches allow for low-cost coatings with good coverage of complex surfaces; however, they may present limitations in terms of crystalline phase control, mechanical adhesion, and reproducibility [72,73].

On the other hand, physical techniques have gained prominence due to their ability to produce high-quality coatings with enhanced adhesion. These include plasma spray (PS), radiofrequency magnetron sputtering (RF-MS), pulsed laser deposition (PLD), and ion beam assisted deposition (IBAD) [73], as well as more recent methodologies such as pulsed electron deposition (PED) [74], matrix-assisted pulsed laser evaporation (MAPLE) [75], and ionized jet deposition (IJD) [8,36]. Additional, less widely adopted techniques include cathodic arc deposition [76] and high power impulse magnetron sputtering (HiPIMS) [77], which, however, face greater challenges in maintaining the stoichiometry of the target material.

These techniques are based on the physical ablation or vaporization of the source material, by means of laser beams, electron beams, ionized plasmas or ion beams followed by condensation of the material as a thin film on the substrate. Compared to chemical processes, physical methods provide improved control over the microstructure, chemical composition, and mechanical properties of the coating, albeit at the expense of higher system complexity and operational costs [73]. Generally, coatings produced with these PVD technologies are less crystalline and more amorphous compared to those obtained through wet depositions; however, post-deposition thermal treatments can increase the degree of crystallinity.

The principal properties and characteristics of the different coating fabrication techniques are reported in Table 2.

### 4.1. Physical Deposition Techniques

#### 4.1.1. Plasma Spray

**Figure 1:**
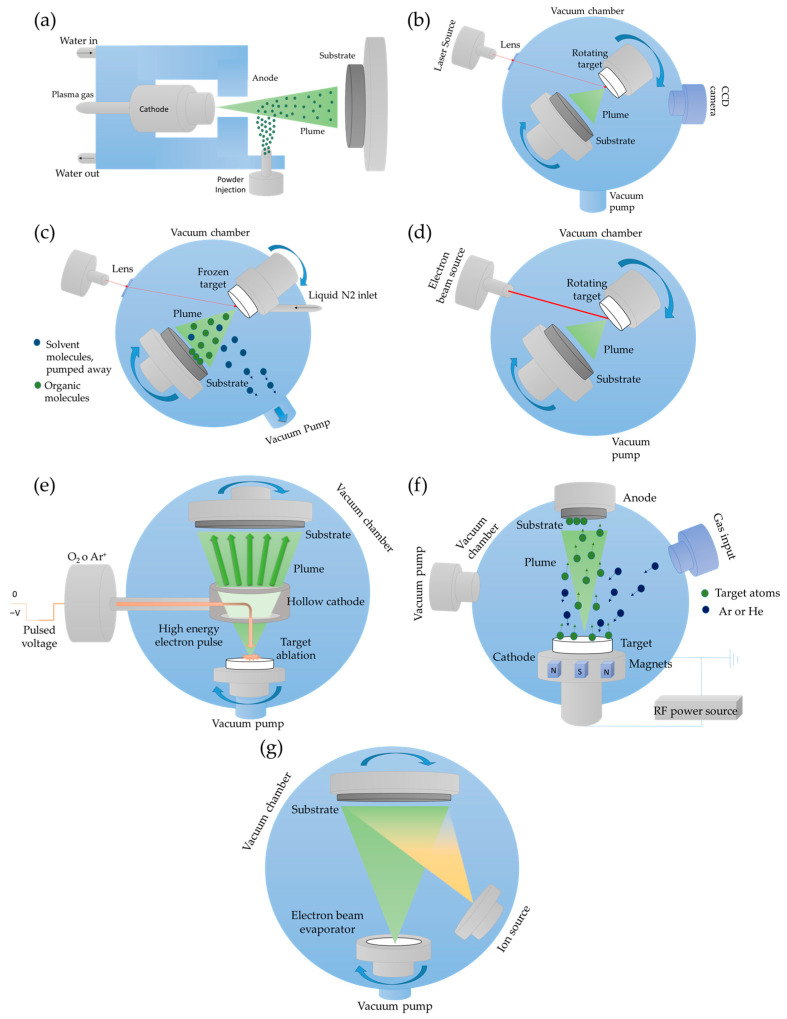
Scheme of the different physical deposition techniques. (**a**) Plasma spray. (**b**) Pulsed laser deposition. (**c**) Matrix-assisted pulsed laser evaporation. (**d**) Pulsed electron deposition. (**e**) Ionized jet deposition. (**f**) Radio frequency magnetron sputtering. (**g**) Ion beam assisted deposition.

Among the various deposition techniques approved by U.S. FDA, plasma spraying stands out from alternative methods for biomedical coatings, due to its superior performance. This technique is particularly suitable for producing thick coatings on components with regular geometries, especially those involving high-temperature CaP phases. Plasma spraying encompasses several technological variants: Atmospheric plasma spraying (APS), vacuum plasma spraying (VPS), solution precursor plasma spraying (SPPS), and suspension plasma spraying (SPS), which differ in operating conditions and obtained morphology. Among these, APS is the most widely adopted method due to its cost-effectiveness, high deposition rate, and capability to coat large surface areas. In APS, a high-frequency electric arc between a tungsten cathode and an anode ionizes a process gas (typically He, H_2_, or N_2_), generating a plasma plume that can reach temperatures up to 16,000 °C. The injected powder feedstock melts rapidly within this plume and is propelled toward the substrate, where it solidifies instantly, forming overlapping lamellae [69,80]. The scheme of the PS deposition technique is reported in Figure 1a.

**Figure 2:**
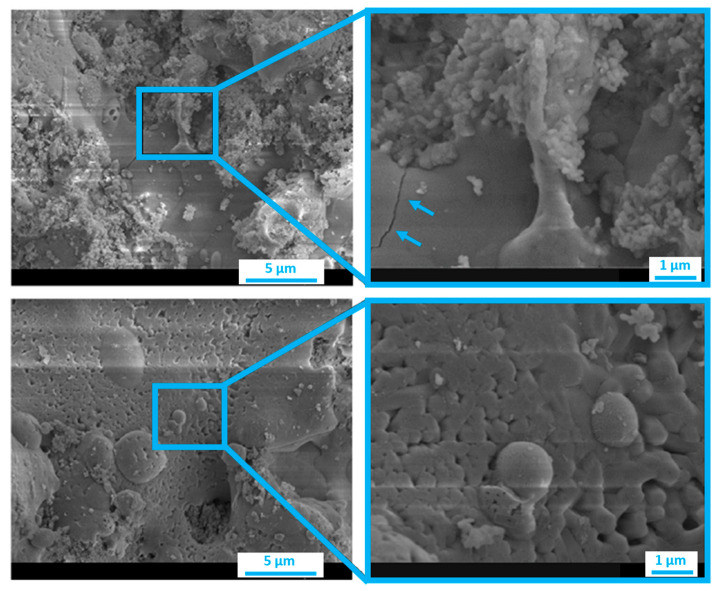
SEM images of hydroxyapatite plasma-sprayed coatings with morphology of molten microdroplets and partially fused spheroidal particles. In addition, the blue arrows underline the cracks present in the hydroxyapatite plasma sprayed coating. Reprinted/adapted from reference [109], licensed under CC-BY 4.0.

The extreme conditions of the plasma jet critically influence coating thickness, morphology, crystallinity, and both mechanical and biological properties. Plasma-sprayed coatings typically reach thicknesses between 30 and 200 μm. However, such dimensions may introduce drawbacks, including crack formation and delamination under mechanical stress, mainly due to residual stresses and internal porosity (Figure 2) [1,15,69,102]. Moreover, the high thermal input required for melting the particles can cause partial decomposition of the feedstock, altering its chemical composition, particularly the ideal Ca/P ratio (1.67), and reducing its resistance to resorption in physiological environments [73]. Morphologically, plasma-sprayed coatings exhibit rough, irregular surfaces with characteristic features such as molten microdroplets, partially fused spheroidal particles (Figure 2). This surface roughness promotes cell adhesion but contributes to heterogeneity in coating-substrate bonding strength [69,103]. Plasma-sprayed CaP coatings are generally characterized by poor tensile strength, with adhesion values often below 35 MPa, wear resistance and fatigue behavior [78,104]. Optimizing adhesion requires fine control of the heat transfer between the plasma and particles, but also promotes hydroxyapatite (HA) decomposition. Strategies to improve adhesion include the introduction of intermediate oxide layers, such as TiO_2_, between the substrate and coating. These layers act as thermal barriers and enhance both mechanical interlocking and chemical bonding. For instance, thermal oxidation of Ti6Al4V substrates at 800 °C has been shown to improve HA crystallinity (from 64% to 75%) and adhesion strength (from 25.9 MPa to 30.7 MPa). Additionally, incorporating additives such as MgO or SiO_2_ into HA coatings has yielded promising biological outcomes, enhancing osteoblast proliferation and differentiation by increasing metabolic activity and alkaline phosphatase expression [11,105].

Overall, plasma-sprayed coatings exhibit excellent osteoconductive behavior [10]. Histological and microscopic analyses have confirmed direct bone apposition on coating surfaces, with no fibrous tissue interposition [106]. Furthermore, these coatings improve the corrosion resistance of metallic substrates by limiting ion release and thereby extending implant longevity [107,108].

#### 4.1.2. Pulsed Laser Deposition

Pulsed laser deposition (PLD) is a physical vapor deposition technique widely employed to produce thin films of materials with high technological relevance, such as hydroxyapatite (HA) for biomedical applications [110]. In this process, the target material, typically a solid, is placed inside a vacuum chamber or in a controlled background gas environment and is irradiated by a high-energy pulsed laser beam, such as excimer lasers (ArF, KrF, XeCl) or solid-state Nd:YAG lasers [34,80]. The laser–target interaction, with the target continuously rotated to ensure uniform ablation, generates a plasma plume rich in atoms, ions, and molecules that deposit onto the substrate to form the coating. Each laser pulse deposits a film thickness in the range of 0.02–0.05 nm, allowing coatings to be fabricated from approximately 50 nm to several microns in thickness [1,15,111]. Precise control of deposition parameters, including laser fluence, target–substrate distance, and substrate temperature, enables tailoring of the film characteristics [112]. Lower substrate temperatures generally yield amorphous coatings, whereas heating the substrate between 350 °C and 600 °C promotes high crystallinity, with the amorphous-to-crystalline transition occurring around 340 °C [1,15,111]. Post-deposition thermal treatments can further enhance crystallinity [1,111]. The scheme of PLD technique is reported in Figure 1b.

**Figure 3:**
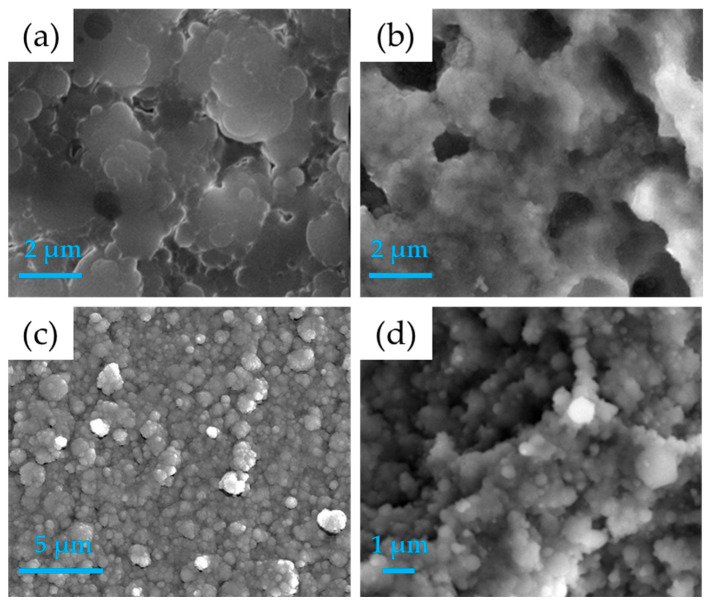
Different SEM images obtained on HA coatings realized with different PVD techniques: (**a**) pulsed laser deposition, (**b**) matrix-assisted pulsed laser evaporation, (**c**) pulsed electron deposition, and (**d**) ionized jet deposition. Images (**a**,**b**) reprinted with permission from reference [119], Copyright 2016 from Elsevier B.V.; (**c**) unpublished data and (**d**) reprinted from reference [8], licensed under CC-BY 4.0.

Morphological observations by SEM often reveal rough and irregular surfaces [113], although the choice of laser significantly influences coating morphology: excimer lasers tend to produce columnar structures, while Nd:YAG lasers yield more granular and mechanically robust morphologies (Figure 3a) [83,114]. Importantly, PLD allows faithful preservation of the target stoichiometry in the deposited films. The Ca/P ratio can be maintained close to the ideal value of 1.67 when operating at an oxygen pressure of 0.1 Torr. Variations in ambient pressure, however, can shift the Ca/P ratio between 1.77 and 2.01, consequently affecting the coating properties [84]. Mechanically, PLD coatings exhibit hardness values ranging between 2 and 3 GPa and an elastic modulus of 80–90 GPa [85]. Amorphous films tend to be more brittle, whereas crystalline coatings, particularly those fabricated with Nd:YAG lasers, demonstrate improved fracture resistance [115]. In vivo studies have shown a significant enhancement in the mechanical strength of implants coated with nanostructured films, nearly doubling that of uncoated titanium implants [11]. Adhesion tests, including pull-off and scratch methods, report that PLD coatings exhibit adhesion strengths exceeding 58 MPa, markedly superior to those achieved via plasma spraying [116].

From a biological standpoint, HA coatings deposited by PLD show excellent biocompatibility, stimulating osteoblast proliferation and promoting osteointegration [83,117,118]. An additional advantage is the controlled dissolution of HA films under physiological conditions, which supports gradual integration with bone tissue [83]. Notably, HA and α+β-TCP coatings on Ti-6Al-4V substrates have been shown to promote stable adhesion of mineralized bone matrix in osteogenic cell cultures derived from rat bone marrow [114].

#### 4.1.3. Matrix Assisted Pulsed Laser Evaporation

The matrix-assisted pulsed laser evaporation (MAPLE) technique is one of the most refined methods for thin film deposition. Unlike conventional PLD, MAPLE uses a frozen target composed of a diluted solution of the material of interest dispersed in a volatile matrix. When irradiated with pulsed laser beams, the energy is primarily absorbed by the matrix, causing a localized temperature to increase that triggers the mechanical vaporization of the dispersed molecules without inducing photolytic or pyrolytic damage [75,120]. The resulting gas phase enables the formation of uniform coatings on substrates placed parallel to the target, which can be heated to controlled temperatures if needed [121]. Thanks to the ability to finely tune parameters such as laser fluence and number of pulses, MAPLE allows precise control over film thickness, typically ranging from 50 to 400 nm depending on operational conditions. The resulting films generally exhibit a particulate or slightly porous morphology, which may enhance cell adhesion phenomena. Surface roughness values typically fall between 100 and 200 nm, with a slight tendency to increase with rising laser fluence (Figure 3b) [86,122]. The scheme of the MAPLE deposition technique is reported in Figure 1c.

From a compositional standpoint, MAPLE enables nearly stoichiometric transfer of precursor materials, ensuring that the Ca/P ratio in hydroxyapatite-based coatings remains close to the ideal value of 1.67. Moreover, the crystallinity of the films can be modulated: typically, coatings display an amorphous or nanocrystalline structure, depending on the post-deposition thermal treatment applied [87].

Biologically, coatings fabricated via MAPLE have demonstrated excellent biocompatibility. For example, hydroxyapatite and silk fibroin-based films supported the adhesion, spreading, and normal morphology of SaOs2 osteosarcoma cells cultured for 72 h [87]. Furthermore, HA coatings functionalized with alendronate promoted osteoblastic differentiation and inhibited osteoclastic proliferation [88]. The use of composite materials such as PHBV/HA has further expanded MAPLE’s potential in bone tissue regeneration, promoting both the growth and osteogenic differentiation of mesenchymal stem cells [122]. MAPLE has been successfully applied to synthesize thin films of alendronate-modified hydroxyapatite on titanium substrates, using composite nanocrystals with varying bisphosphonate content. The resulting thin films exhibited good crystallinity, which slightly decreased with increasing alendronate content, while maintaining a characteristically porous structure. In vitro biological tests confirmed the bioactivity of the coatings: MG63 osteoblastic cells cultured on these films displayed normal morphology, enhanced proliferation, and high levels of differentiation markers such as type I collagen and osteocalcin [88,123,124].

#### 4.1.4. Pulsed Electron Deposition

Pulsed electron deposition (PED) is emerging as a valid alternative to pulsed laser deposition (PLD), offering a more cost-effective and efficient approach for thin film synthesis. In contrast to PLD, where ablation is induced by a laser beam, PED employs high-energy pulsed electron beams to generate a plasma plume from the target material. The beam generation system consists of a hollow cathode connected to a high-voltage generator, a ring-shaped anode, and a dielectric tube that connects and extends beyond the anode. By applying a potential difference of up to 20 kV, electrons are accelerated toward the target, triggering the emission of a highly ionized plasma. The underlying mechanism is similar to that of PLD, but the distinct ablation source grants PED several key advantages: notably, a higher pulse energy frequency, superior energy efficiency, and the ability to ablate materials with high reflectivity or wide bandgaps [74]. The scheme of the PED technique is reported in Figure 1d.

From a coating perspective, PED-deposited films are composed of globular aggregates with sizes ranging from 20 to 80 nm and exhibit a highly rough surface morphology, with average roughness (Ra) in the range of 100–200 nm (Figure 3c) [89]. This surface roughness is particularly favorable for cell interactions, promoting adhesion, proliferation, and the commitment of host cells, toward osteogenic differentiation [90].

Film thickness can be precisely controlled during the PED process, typically ranging from 300 to 800 nm. CaP coatings deposited by PED are generally amorphous at the end of the deposition process. However, their crystallinity can be enhanced through appropriate post-deposition thermal treatments, enabling tuning based on specific application needs. Furthermore, the Ca/P ratio of the films can be tailored to closely resemble that of biological hydroxyapatite [125].

In vitro biological assays have confirmed the excellent biocompatibility and bioactivity of PED coatings, attributable to their nanostructured surface morphology and the capacity to support cell proliferation without inducing cytotoxic effects [90].

#### 4.1.5. Ionized Jet Deposition

A significant evolution of pulsed electron deposition (PED) is represented by ionized jet deposition (IJD), a technique based on a pulsed electron source capable of generating ultra-short electrical discharges in vacuum, reaching the megawatt range. In particular, the IJD is a specific setup of pulsed electron deposition and differs from PLD mainly because it uses an electron beam to ionize the sputtering target material, unlike the laser beam used in PLD. Furthermore, compared to PED, IJD employs auxiliary electrodes and trigger system to make current transmission to the target more effective. These discharges are produced via high-voltage pulses (up to 25 kV) with a duration shorter than one microsecond. The system is assisted by a gas jet that supports the discharge and by a solid target, toward which energy is directed through a trigger system and auxiliary electrodes. This setup leads to a surface explosion of the target, resulting in a plasma plume that subsequently deposits onto the substrate [74]. The scheme of the IJD technique is reported in Figure 1e.

From a morphological perspective, coatings obtained via IJD are dense and exhibit a nanostructured surface with controllable roughness, generally in the range of hundreds of nanometers [93,126]. The surface morphology typically consists of nanometric grains (~80 nm) aggregated into clusters up to 1.5 µm in size (Figure 3d) [8,36,127]. Moreover, the technique allows for a highly accurate preservation of the target’s chemical composition, which is particularly beneficial in coatings based on biogenic apatites: the Ca/P ratio can be maintained close to that of natural hydroxyapatite, while also retaining biofunctional minor ions such as Mg and Na Films deposited via IJD are initially amorphous, but can be partially crystallized through controlled thermal treatments (e.g., 400 °C for 1 h) [128,129]. Adhesion to the substrate is also excellent, thanks to the possibility of optimizing deposition parameters and performing post-deposition treatments. This has been demonstrated on both metallic substrates like titanium and polymeric materials such as PEEK [90].

From a biological standpoint, films derived from biogenic targets have shown superior bioactivity compared to synthetic hydroxyapatite coatings [90]. The nanostructure and biomimetic composition of IJD coatings can guide cellular behavior, stimulating adhesion and proliferation [8,130], and influencing mesenchymal stem cell morphology [62,131], thereby supporting osteogenic differentiation [62]. Furthermore, IJD has proven effective in fabricating antibacterial coatings, for example through the deposition of nanostructured silver (Ag) films that combine antimicrobial properties [132] with stem cell compatibility [133].

#### 4.1.6. Magnetron Sputtering

Magnetron sputtering (MS) is a widely used physical vapor deposition (PVD) technique for the fabrication of thin films. This process employs a plasma, typically of argon, neon, krypton, or xenon to eject atoms from a negatively charged target, which are then deposited onto a substrate within a vacuum chamber [80]. Several parameters influence the quality and integrity of CaP-based coatings produced by sputtering, including discharge power, gas flow rate, working pressure, substrate temperature, deposition time, post-deposition thermal treatments, and the possible application of a negative bias to the substrate [1]. A particularly effective configuration is radio frequency (RF) MS, where the target surface is RF polarized, enhancing ionization efficiency. In this mode, the target material is ionized under vacuum using negatively charged magnets, enabling efficient material transfer to the substrate. While direct current (DC) sputtering is suitable for conductive materials, RF sputtering is generally preferred for CaP deposition due to the insulating nature of many calcium phosphate compounds. The scheme of the RF-MS deposition technique is reported in Figure 1f.

RF magnetron sputtering offers several advantages, including high deposition efficiency, the ability to coat heat-sensitive substrates, excellent control over film thickness (typically ranging from 40–50 nm to 3.5 µm), uniformity, and strong adhesion to the substrate (bonding strength > 30 MPa) [15]. Higher adhesion strengths for films deposited by sputtering are reported compared to those produced by plasma spraying [79]. Studies have shown higher values: hydroxyapatite (HA)/Ti coatings on Ti–6Al–4V substrates achieved by RF magnetron sputtering demonstrated adhesion strengths between 60 and 80 MPa [134]. Despite these benefits, sputtering also presents limitations, such as high equipment costs and relatively low deposition rates [79]. Coatings obtained via MS are generally amorphous without post-deposition thermal treatments, dense, and pore-free, even at low deposition temperatures [135]. However, the fabrication of highly oriented HA thin films by magnetron sputtering is also reported [82]. The nanohardness and Young’s modulus of such coatings typically reach 10 and 100 (GPa), respectively. Chemically, the Ca/P ratio in sputtered films can vary widely (1.6–2.6), often exceeding the stoichiometric value of hydroxyapatite (1.67) [15].

Biologically, the deposition of carbonated hydroxyapatite via RF magnetron sputtering has produced films without cytotoxic effects on host cells. In vitro studies have demonstrated suitable adhesion, viability, and proliferation of osteoblasts on these coatings. Furthermore, after 21 days of immersion in simulated body fluid (SBF), mineralization was observed along with the differentiation of mesenchymal stem cells into osteoblast-like cells [26,81,136]. In another study, a thin HA film (500 ± 20 nm) was successfully deposited on an AZ91 magnesium alloy using RF magnetron sputtering, resulting in a significant improvement in the substrate’s corrosion resistance [137].

#### 4.1.7. Ion Beam Assisted Deposition

Ion-beam-assisted deposition (IBAD) is a vacuum-based technique particularly suitable for the fabrication of thin ceramic coatings on a wide range of substrates, including metals, polymers, and ceramics [138,139,140]. In the IBAD process, physical vapor deposition is combined with the simultaneous bombardment of the growing film surface using a broad-beam ion source operating at low pressure [80]. The energy delivered by the ions actively modifies both the substrate and the coating during growth, promoting strong adhesion and the formation of an intermixed interface layer. Key parameters influencing the microstructure and chemical composition of the coating include the evaporation rate, the nature of the target material, ion species and energy, and ion current density [80,91]. The scheme of IBAD technique is reported in Figure 1g.

From a physical standpoint, CaP coatings obtained by IBAD can range in thickness from a few angstroms to several microns, typically between 2 and 4 µm [91,141]. The resulting films are dense, continuous, and free of pores or visible cracks, while also preserving the native roughness of the underlying substrate [1]. The elemental composition, particularly the Ca/P ratio, can be finely tuned during deposition [131]. Moreover, the CaP coating obtained through IBAD process has a higher average hardness (6.4 GPa) and Young’s modulus (132 GPa) as compared to both sputter-deposited and sintered HA. This better integrity circumvents issues commonly associated with post-deposition thermal treatments, such as crack formation due to thermal expansion mismatch between the substrate and coating, which are frequently observed in plasma-sprayed films [80,92].

Biologically, IBAD-derived coatings have demonstrated excellent in vitro and in vivo performance. Studies report no cytotoxicity to host cells, along with good cell adhesion, osteoblastic viability and proliferation, and effective mineralization in simulated body fluids [80,139].

### 4.2. Chemical Deposition Techniques

In the field of bone implant coatings, physical deposition techniques for CaP-based coatings present several intrinsic limitations: they operate under non-physiological conditions, often at high temperatures or in high vacuum environments. Additionally, the use of complex and expensive equipment limits their scalability and large-scale adoption. Considering these drawbacks, there is growing interest in wet-chemical techniques, which offer a simpler, more cost-effective, and scalable alternative. These approaches are based on solution-phase chemical processes and are particularly suitable for coating complex surfaces and porous structures, such as three-dimensional scaffolds used in tissue engineering. Among the most studied wet-chemical methods are biomimetic deposition, sol-gel, and electrodeposition. These techniques allow not only greater versatility in the choice of substrates, but also finer control over the coating composition, morphology, and crystalline structure [72,142,143].

#### 4.2.1. Biomimetic Deposition

**Figure 4:**
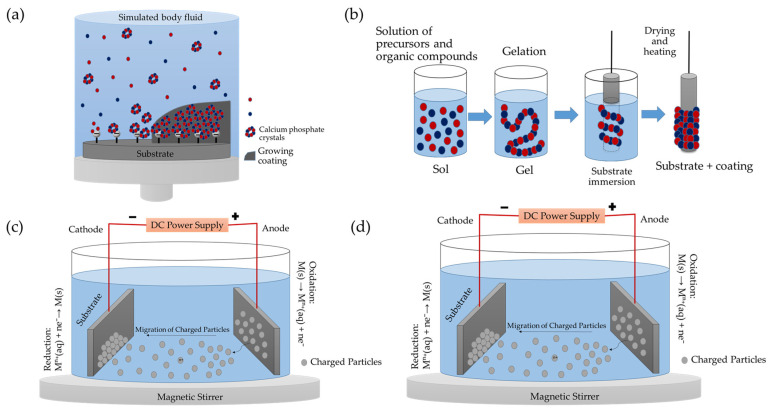
Scheme of the different chemical deposition techniques. (**a**) Biomimetic deposition. (**b**) Sol-gel deposition. (**c**) Electrophoretic deposition. (**d**) Electrochemical deposition.

**Figure 5:**
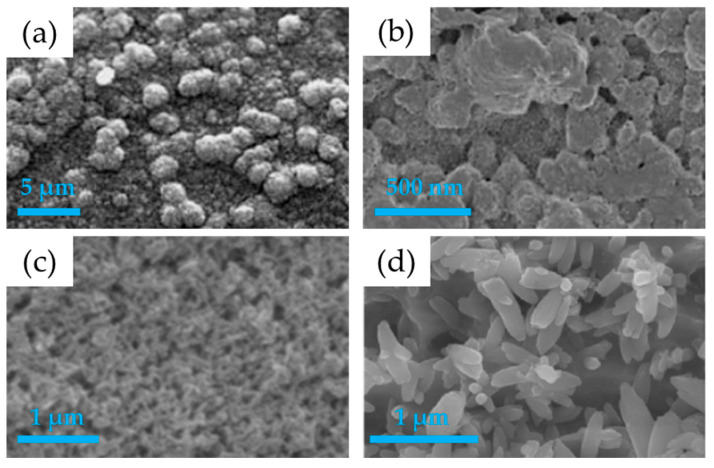
SEM images obtained on HA coatings prepared with different chemical deposition techniques: (**a**) biomimetic deposition, (**b**) sol-gel deposition, (**c**) electrophoretic deposition and (**d**) electrochemical deposition. Images reprinted/adapted (**a**) with permission from reference [146], Copyright 2005 from Elsevier; (**b**) from reference [154], licensed under CC-BY 4.0; (**c**) from reference [155], licensed under CC-BY 4.0, and (**d**) from reference [156], licensed under CC-BY.

Biomimetic deposition (BD) is among the most promising techniques for coating implant surfaces with CaPs, due to its ability to replicate physiological conditions of the human body during the deposition process. Introduced by Kokubo in 1990 [144], this method involves immersing a pre-functionalized substrate in a supersaturated solution of calcium and phosphate ions (simulated body fluid, SBF). SBF is formulated to match the ionic composition, pH (approximately 7.4), and partial pressure of CO_2_ (0.05 atm) of human plasma. The process begins with surface modification of the substrate, which may involve the application of functional groups (such as –OH, –COOH) or CaP seeds that serve as nucleation sites for apatite precipitation [63]. Once immersed in the SBF, the substrate promotes the spontaneous formation of a CaP coating through chemical precipitation, a mechanism that mimics the physiological mineralization of bone matrix [96,145]. The resulting coating is composed of a nanocrystalline carbonated apatite phase, morphologically and chemically similar to natural bone tissue. An example of biomimetic deposition system is reported in Figure 4a. A fast biomimetic deposition method on Ti6Al4V substrates using a slightly supersaturated CaP solution with an ionic composition simpler than that of SBF was later developed (Figure 5a) [146]. At variance with other fast deposition methods, which produce amorphous calcium phosphate coatings, the new proposed composition allows one to obtain nanocrystalline HA.

The thickness of the film is difficult to precisely control due to the spontaneous nature of the growth process. It can range from a few hundred nanometers to several micrometers, depending on the immersion time, which may vary from several days to weeks, and on the solution conditions. Surface roughness, typically between 100 and 300 nm, is favorable for cell proliferation. Although adhesion may be weak if the substrate is not properly pretreated, it can be improved by increasing surface roughness. The morphology is generally flake-like or flower-like, with a porous structure that enhances interaction with biological fluids [97]. The Ca/P ratio tends to approach the stoichiometric value of hydroxyapatite (1.67) but may vary depending on the ions present in the solution and the degree of crystallinity. The latter, initially low, can increase with prolonged immersion times or post-deposition treatments [147]. A major strength of biomimetic technique is the ability to incorporate functional ions such as Mg^2+^, Sr^2+^, or Mn^2+^ into the coating, which modulate crystal growth and improve the biological properties of the coating [98].

Coatings obtained through this technique have demonstrated high biocompatibility, osteoconductivity, and, in some cases, osteoinductive potential in both in vitro and in vivo studies [27,148,149]. Additionally, co-deposition of biomolecules during apatite formation allows for targeted functionalization of the coating: molecules such as osteocalcin [150], fibronectin [151], or bioactive polymers like poly(L-lysine) [81], can be effectively integrated into the mineral matrix. Notably, the BMP-2 protein, essential for inducing osteogenesis, can be incorporated and released in a controlled manner from the coating, stimulating the local formation of new bone tissue [152].

#### 4.2.2. Sol-Gel Deposition

The sol-gel deposition technique is one of the most versatile and straightforward methods for producing thin ceramic oxide coatings and is widely employed for the deposition of hydroxyapatite layers on metallic substrates used in implantology. The process is based on a sequence of chemical reactions that start with soluble precursors in solution and lead to the formation of a “sol,” which gradually evolves into a “gel” through polycondensation. Typically, the initial solution contains salts such as CaCl_2_ and Na_3_PO_4_, which provide the calcium and phosphate ions required for apatite synthesis [80]. Once the gel is formed, it is deposited onto the substrate using methods such as dip-coating, spin-coating, or spraying. After deposition, the film is subjected to moderate thermal treatments (typically between 300 and 600 °C) to remove organic residues and induce crystallization of the coating, while preserving a certain degree of porosity and a microstructure that is compatible with biological environments. The scheme of the sol-gel deposition technique is shown in Figure 4b.

The hydroxyapatite formed by sol-gel often exhibits a nanocrystalline structure, with crystal morphologies ranging from platelets to circular petal-like clusters (Figure 5b). The Ca/P ratio typically approximates the stoichiometric value of HA (1.67), although it may vary slightly depending on the synthesis parameters [94]. A key aspect of sol-gel coatings is their tunable roughness, which can be controlled by adjusting deposition parameters and precursor chemistry. This feature allows good adhesion to the substrate, with bond strengths exceeding 30 MPa in some cases. An optimal thickness below 1 µm is generally recommended to ensure uniform and defect-free films, because excessively thick coatings may lead to cracking due to shrinkage during the drying phase.

From a biological standpoint, HA coatings obtained via sol-gel exhibit good bioactivity [95]. Nevertheless, their high porosity can result in an accelerated dissolution rate in SBF, potentially compromising the long-term stability of the coating and its integration with bone tissue [80]. To mitigate this effect, strategies such as the co-deposition of stabilizing oxides (e.g., TiO_2_, ZrO_2_) or the use of hybrid matrices incorporating biodegradable polymers like polycaprolactone have been explored [153].

#### 4.2.3. Electrophoretic and Electrochemical Deposition

Two of the main techniques employed for the fabrication of ceramic coatings are electrophoretic deposition (EPD) and electrochemical deposition (ECD), both of which rely on the application of electric fields to promote the formation of ceramic films on substrate surfaces.

Electrophoretic deposition is based on the principle that electrically charged ceramic particles, such as hydroxyapatite (HA) nanoparticles, suspended in a liquid medium, migrate toward a conductive substrate under the influence of an externally applied electric field [99]. The most commonly used dispersing media are organic solvents like ethanol or isopropanol, which help stabilize the suspension [157]. EPD is appreciated for its simple setup, low cost, and the ability to coat even complex geometries uniformly, with coating thicknesses ranging from less than 1 μm to over 100 μm [80,100]. The mechanism of EPD is displayed in Figure 4c. A key advantage of EPD is the ability to obtain homogeneous coatings of high purity and controlled stoichiometric composition, regardless of the shape of the substrate [99]. However, a known limitation is the formation of cracks due to shrinkage during post-deposition sintering. This step is critical, as it involves high temperatures that can affect both the metallic substrate and the coating itself [100]. The morphological properties of the coating vary according to the shape of the particles employed. Coatings derived from nanoparticles with regular, equiaxed morphology, such as spherical or rounded shapes, exhibit greater resistance to crack formation (Figure 5c) [11]. Surface roughness, which can be controlled through deposition parameters and particle size distribution, plays a key role in cell adhesion and tissue integration. Under optimized conditions, EPD coatings have shown mechanical adhesion to the substrate in the range of 50–60 MPa, which is higher than that of coatings obtained via plasma spray techniques [158].

Recent studies have demonstrated that the application of a dynamic voltage during electrophoretic deposition allows the fabrication of gradient-structured hydroxyapatite coatings, with a dense, adherent inner layer and a more porous outer layer. This configuration enhances both mechanical adhesion and bioactivity, improving interaction with bone cells [159].

The biological performance of these coatings has been widely investigated. In vitro tests have shown that EPD coatings support good biocompatibility, promoting cell proliferation and osteoblast adhesion on the coating surface [11]. The nanostructured morphology of the apatite, combined with the presence of components such as titanium oxide [160] and carbon nanotubes [161,162], has proven effective in promoting bone tissue bonding and enhancing implant performance even in challenging environments [157].

Alongside EPD, electrochemical deposition represents a promising alternative for applying bioactive coatings to metallic substrates (Figure 4d). Unlike EPD, where pre-formed particles deposit through electrophoretic migration, electrochemical deposition is based on the controlled decomposition of an electrolyte solution containing calcium and phosphate ions, triggered by an applied electric field [72,101]. This process typically occurs at the cathode, where the generation of OH^−^ ions causes a local increase in pH, initiating the nucleation and direct growth of a calcium phosphate layer on the metal surface. Among its main advantages are high adhesion strength of the film to the substrate, the possibility to achieve a Ca/P ratio close to that of stoichiometric HA, average coating thickness around 200 nm, and tunable morphology through electrochemical parameters such as current density, electrolyte composition, and pH (Figure 5d). Cell culture experiments have shown that CaP-coated surfaces are non-toxic to osteoblastic cells in vitro and can support cell growth [163,164]. Furthermore, the process can be conducted at room temperature, making it suitable for heat-sensitive substrates and avoiding the post-deposition sintering step typically required in EPD [165].

## 5. CaP Coatings Application in Orthopedic Fields

**Figure 6:**
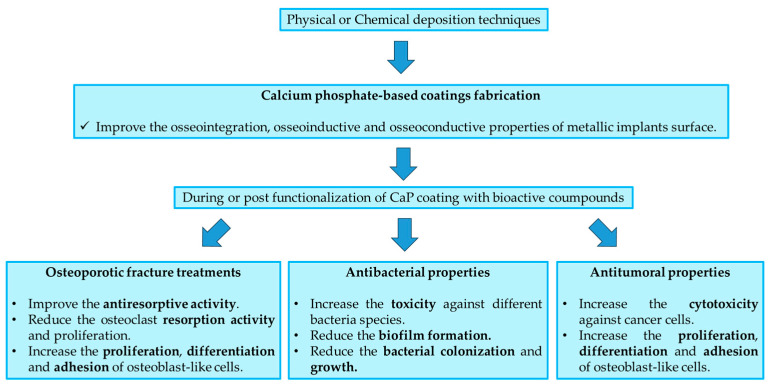
Possible application of CaP coatings functionalized with bioactive compounds in orthopedic applications.

Micro- and nano-structured coatings are used to increase the performance of biomedical devices and metallic prosthesis in dentistry and orthopedics. In particular, the principal macro-areas of applications consist in (i) increase the osseointegration and osseoinductive features of inert metallic prosthesis surfaces, (ii) increase the antibacterial properties to avoid the bacteria contamination and biofilm formation on their surface, and (iii) increase the anti-tumoral effects against the tumor cells remained after cancer elimination (Figure 6). The CaP coatings, as mentioned before, modify the surface chemistry following the biomimicry; however, the coupling with specific bioactive compounds and drugs can improve the therapeutic efficacy regarding the three macro-areas reported.

### 5.1. Osteoporotic Fracture Treatments—Osseointegration Properties

Osteoporosis is one of the major bone pathologic conditions diffused in the world-wide population regarding the bone tissue. In general, osteoporosis is connected to a metabolic skeletal disorder which causes a bone mass loss and accelerated destruction of bone microstructure [166,167]. Osteoporosis is diagnosed when the bone mineral density of the patient is 2.5 below the adult mean [167,168]. The reduction in bone mass mainly causes bone frailty and subsequent fractures [168]. The bone fractures are concentrated at the femur neck and the strategy to restore a physiological condition at the structural and biomechanical level is to apply a metal prosthetic device (mainly titanium alloy). However, an important aspect lies in reducing and avoiding the course of the disease that could lead to further fractures. There are several therapies used to reduce the worsening of the osteoporotic condition in patients; the avenues taken can broadly be divided into two: diet modification and drug administration (anti-resorptive and anabolic drugs). Vitamin D represents an important element involved in the bone health and phosphorous and calcium metabolism, and its alteration and deficiency can alter the correct physiology of human bone tissue [169], causing the osteoporotic condition. Current strategies focus on diet, use of vitamin D, calcium and protein intake to fight the progression of the osteoporotic condition [170]. Regarding the drug administration, anti-resorptive agents such as selective estrogen receptor modulators (SERMs), strontium ranelate, estrogen replacement therapy (ERT) and bisphosphonates (BPs) are the most diffused strategies to avoid and reduce the bone resorption rate [170]. In addition, the use of some growth factors such as bone morphogenic protein 2 and 4 (BMP2, BMP4), fibroblast growth factor, VEGF, and platelet-derived growth factor can favor new bone growth and regeneration [171].

Also, vitamin C is used to improve the osteoblast cell proliferation and reduce the osteoclastogenesis. In fact, Majumdar U. et al. have realized hydroxyapatite-coated titanium implants by the plasma spray technique and, after that, they have loaded the coating realized by drop-casting with vitamin C and curcumin to improve biological features. The coating created demonstrated cytocompatibility against human osteoblast cells, reducing in osteosarcoma cell viability and antibacterial efficacy against *S. aureus* [172].

**Figure 7:**
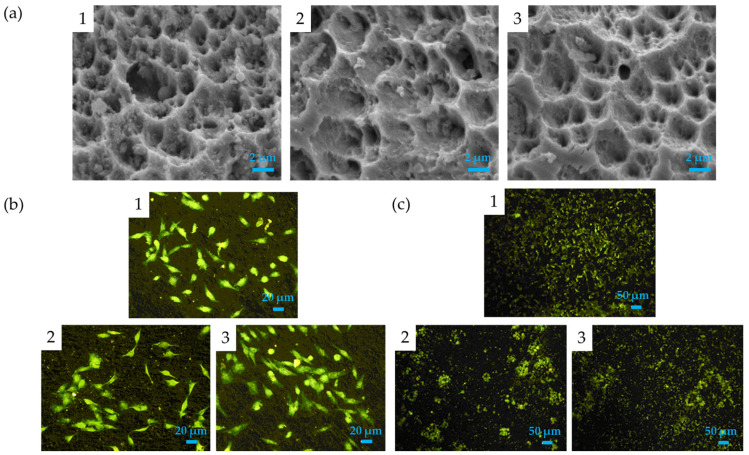
Coatings realized with MAPLE deposition to increase the anti-resorption properties of titanium surface implants. In particular, hydroxyapatite (1), alendronate-hydroxyapatite (2, alendronate 7 mm) and alendronate-hydroxyapatite (3, alendronate 28 mm) are reported. (**a**) SEM images of the different coating surfaces are reported (scale bar: 2 µm). (**b**) Phalloidin staining of osteoblast-like cells cultured for 24 h (scale bar: 20 µm). (**c**) Phalloidin staining of osteoclast cells cultured for 24 h (scale bar: 50 µm). Images reprinted/adapted with permission from reference [88], Copyright 2005 from Elsevier.

Another material used for prosthesis fabrication is polyetheretherketone (PEEK) and the application of alendronate hydroxyapatite nanoparticles coupled with subsequent interleukin-4 grafting was investigated to increase the osteoimmunomodulation and bone regeneration properties. The release of calcium ions and alendronate improved osteogenesis and reduced the osteoclastogenesis, while IL-4 created an osteoimmunomodulation microenvironment due to its ant-inflammatory properties [173]. The fabrication of HA coating by electrophoretic deposition to reduce the inflammation process and favor the osteoimmunomodulation was also reported by Baheti W. et al., where graphene oxide-HA coatings are applied onto titanium implants. The bone marrow mesenchymal stem cell viability and osteogenic differentiation was improved and the M2 macrophage polarization was favored in bringing about an anti-inflammatory environment [174]. Chen M. et al. have applied hydroxyapatite and phosphorylated osteogenic growth peptide onto titanium implants to increase their osteogenic properties on mesenchymal stem cells to improve the treatment of osteoporotic bone fracture repair [175]. Here, the HA coating on Ti surface was obtained by electrochemical deposition and, after that, the phosphorylated osteogenic growth peptide was grafted by chelation. Matrix-assisted pulsed laser evaporation was used to create biofunctional alendronate–HA thin films and HA coatings multifunctionalized with strontium and zolendronate able to promote osteoblast differentiation and to inhibit osteoclast proliferation [88,176]. In particular, alendronate-HA films fabricated with MAPLE deposition using different loading amounts of alendronate (7 and 28 mm) possess a nanostructured morphology, and from phalloidin staining it was possible to notice how the number of osteoclast cells is reduced in the presence of alendronate with regard to the osteoblast number, which remain unaltered (Figure 7).

Thanks to its mild conditions, the MAPLE technique offered the possibility to deposit thin films of OCP at different contents of calcium alendronate on Ti substrates in order to obtain coatings able to offer a suitable interface for bone tissues thanks to the presence of OCP, and to provide a local availability of the bisphosphonate [177]. The osteoclastic activity inhibition by zoledronic acid (ZOL) was also investigated by Zhu M. et al., where previously coated stainless-steel substrate with mesoporous silica nanoparticles combined with HA are loaded with ZOL. The ZOL released by the coating showed a reduction in osteoclast activity by pit formation assay [178].

The application of calcium phosphate-based coating with different bioactive compounds is reported in Table 3.

### 5.2. Antibacterial Properties

In orthopedics and dentistry, implant-related infection is one of the most diffuse problems connected with metallic alloy prostheses and implants and can occur in different post interventions for certain patients, with high impact during recovery. Bacterial contamination can occur in different ways, but principally from blood and at the perioperative stage [180]. The principal bacterial strains involved in orthopedic infections are Gram-positive (*Streptococcus* and *Enterococcus* species) and Gram-negative (*Pseudomonas aeruginosa* and *Escherichia coli*). Antibiotics are the most common treatment, generally gentamicin in orthopedic device infections [180,181,182]. However, the administration of these drugs can cause the development of drug resistant bacteria, such as methicillin resistant *S. aureus* (MRSA) strains [180,181,183,184]. To avoid antibiotic resistance, metal ions (silver, copper, iron, zinc, gold, etc) and nanoparticles can be used due to their ability to reduce bacteria vitality through different biological mechanisms, related to structure of the cell membrane, production of reactive oxygen species (ROS), DNA functions and enzyme or co-factor in some cellular process such as catalytic reactions [185,186].

**Figure 8:**
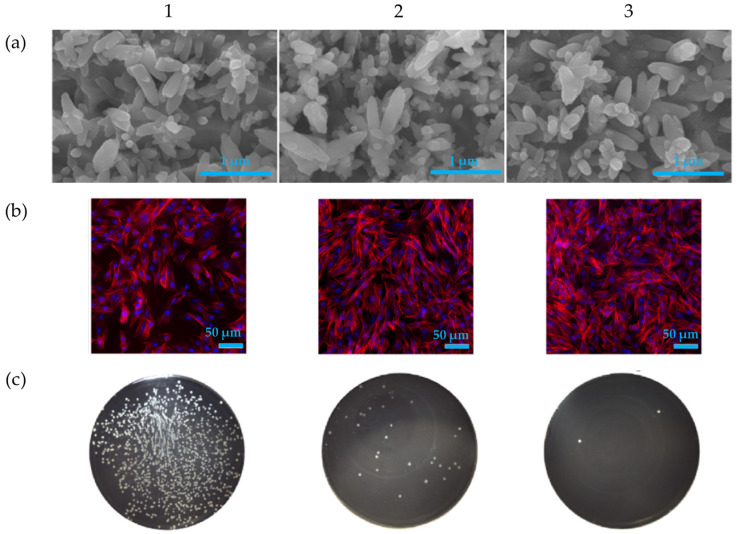
Coatings realized with electrochemical deposition to increase the antibacterial properties of titanium surface implant. In particular, hydroxyapatite (1), low dose simvastatin-hydroxyapatite (2, simvastatin 10^−4^ mol/L) and high dose simvastatin-hydroxyapatite (3, simvastatin 10^−3^ mol/L) are reported. (**a**) SEM images of the different coating surfaces are reported (scale bar: 1 µm). (**b**) Phalloidin and DAPI staining of BMSCs cultured for 24 h (scale bar: 50 µm). (**c**) Spread plate assay on *S. aureus* cultured for 24 h with simvastatin released from coatings. Images reprinted from reference [156], licensed under CC-BY.

Sun T. et al. have obtained hydroxyapatite coatings on titanium alloy implant surfaces and added simvastatin to improve the antibacterial effect against Staphylococcus aureus and Staphylococcus epidermidis by the electrochemical deposition. The coating possessed good biocompatibility on mesenchymal stem cells and avoided the biofilm formation by the different bacteria strains [156]. The authors have used different amounts of simvastatin to load the HA coating during the electrochemical deposition (10^−4^ mol/L—low dose and 10^−3^ mol/L—high dose). The coating morphologies were not altered by the different drug loading. No cytotoxic effects were observed on the bone marrow mesenchymal stem cells in all conditions with promotion in their viability, attachment and proliferation. Regarding the antibacterial effect, a reduction in viable *S. aureus* bacteria was reported on the simvastatin-loaded hydroxyapatite coatings compared with only the HA coating, with a dose-dependent toxic effect reported (Figure 8).

The osteoconductivity properties of the coated implant are also important, as discussed before, so, for this reason, Xie C.M. et al. deposited silver nanoparticles and HA onto titanium surfaces by electrochemical deposition, following which the addition of chitosan and BMP-2 protein was applied to increase the osteoinductivity of implant surface. The combination of chitosan and silver antibacterial properties brought a reduction in both *S. epidermidis* and *E. coli*. Furthermore, BMP-2 promoted BMMSCs differentiation, up-regulating alkaline phosphatase (ALP) production [187]. The loading of antibiotic agents in the coating was also explored by other authors. For example, ampicillin and vancomycin were added by direct absorption to hydroxyapatite nanocoating realized by the hydrothermal method onto a PEEK surface. The antibacterial effect of the mixture realized was tested against *S. aureus* and *E. coli* with an increase of the inhibition area up to 10 days reported [188]. The loading of vancomycin is also explored by Khanmohammadi S. et al., where coatings of mesoporous bioactive glass/hydroxyapatite/chitosan were applied to a titanium surface by electrophoretic deposition. The mesoporous structure of the bioactive glass was used to load vancomycin in the coating with increasing antibacterial properties against *S. aureus* compared to uncoated samples [189]. Vancomycin was also incorporated coupled with chitosan in a hydroxyapatite-coated titanium alloy implant by the plasma spray technique [190,191]. In particular, the porosity of the coating realized was exploded to load chitosan–vancomycin by vacuum impregnation. Due to the only mechanical bonding between the chitosan–vancomycin and HA coating, an initial burst of the drug realized was noticed in the first week with an antibacterial effect against *S. aureus* [191]. Another antibiotic agent used is ciprofloxacin, which was used as loading antibacterial agent in ciprofloxacin-loaded hydroxyapatite coating applied to an NiTi alloy implant by the sol-gel deposition method. Also, in this case, an improvement in *E. coli* bacterial growth was obtained [192]. Hajinaebi M. et al. have loaded ciprofloxacin to nanohydroxyapatite coating electrophoretic deposited by absorption onto the surface, increasing the antibacterial effect against both *S. aureus* and *E. coli* [193]. Some natural compounds such as curcumin and vitamin C have demonstrated increasing antibacterial properties when added to calcium phosphate-based coatings [172]. The application of calcium phosphate-based coatings with different bioactive compounds to increase antibacterial properties are reported in Table 4.

### 5.3. Antitumoral Properties

One of the most diffused strategies consists in the targeting of specific malignant cell receptors involved in cell proliferation and differentiation [194]. In particular, anti-cancer drugs such as doxorubicin, cisplatin, denosumab, bisphosphonates and paclitaxel are used in several clinical applications due to their ability to reduce and fight cancer cell growth [194,195]. In most cases, the development of metastases in bone tissue can lead to osteolytic lesions with increasing risk of fracture and the necessity of surgical intervention with metallic prosthesis application or surgical fixations [196]. The use of metallic prostheses and implants to reconstruct a patient’s specific anatomy of bone defect after bone tumor surgeries is used in the orthopedic field [197,198]. In addition, in some cases, such as vertebral tumors, cancer cause bone fractures, which need the application of a metallic prosthesis [199,200]. In particular, the application of 3D printed metallic porous implants can favor and enhance bone tissue ingrowth due to their osseointegration properties. However, the metal surface is inert and one of the major problems connected with bone surgeries in cancer patients is the slow recovery with daily activity and quality of life implications [199]. The application of calcium phosphate coatings can accelerate the primary stability process between the prosthesis surface and surrounding patient bone tissues.

Li B. et al. fabricated a hydroxyapatite coating on a titanium prosthesis surface by micro-arc oxidation deposition and then coupled this coating with paclitaxel nanospheres to increase the toxicity against tumor cells. The fabricated coating possessed a higher cytotoxic effect against cervical carcinoma cells with respect to osteoblast MC3T3-E1 [201]. Several authors have reported the combination of hydroxyapatite with doxorubicin to increase the antitumoral properties of calcium phosphate-based materials [202,203]. In particular, Zhu X. et al. coupled HA with doxorubicin and small interfering ribonucleic acid (siRNA) and then incorporated into electrospun nanofibrous poly(lactic-co-glycolic acid) (PLGA). The doxorubicin and siRNA showed antitumoral properties against human ovarian cancer cells and human breast cancer cells [204]. Curcumin and epigallocatechin gallate (EGGG) from green tea were applied with drop-casting onto a plasma spray hydroxyapatite-coated titanium implant to enhance bioactivity and induce chemopreventive with viability reducing in MG63 human osteosarcoma cells and proliferation increasing of human fetal osteoblast cells (hFOB) [205]. The interesting anticancer properties of curcumin coupled with calcium phosphate materials is also investigated by Jo K. et al., where the tricalcium phosphate and HA-coated Ti alloy implants were combined with curcumin and vitamin D3, increasing the osteoblast cells (hFOB) viability and cytotoxic effects against MG63 [206]. Iosub G. et al. fabricated a hydroxyapatite coating by MAPLE deposition on titanium samples with the application of carboplatin and quercetin to improve antitumoral protection properties. In fact, the coatings reported good viability and adhesion on hFOB with respect to the MG63 osteosarcoma cells, with their viability was reduced to around 30% [207]. Metal ions commonly used to increase antibacterial effects can also be applied for their intrinsic toxic properties to antitumoral applications. In fact, for example, selenium HA coating applied by micro-arc oxidation can increase the toxic effects against cancer cells, such as epithelial carcinoma cells, and promote the osteogenic differentiation of mesenchymal stem cells [208].

The application of calcium phosphate-based coating with different bioactive compounds is reported in Table 5.

## 6. Clinical Translation of CaP Coated for Orthopedic Implants—Regulatory Point of View

CaP coatings on orthopedic implants are designed to promote the formation of new bone tissue around the implant surfaces, thereby reducing the time required to achieve primary stability of the prostheses. Numerous clinical trials have demonstrated that coatings produced according to standards achieve the desired effect [209,210,211], particularly in total hip and knee prostheses. In the context of biomedical devices, the quality and validation of such coatings are governed by a set of international standards (e.g., ISO, ASTM), which are developed and monitored by regulatory authorities at the global level, such as Notified Bodies or the EMA (European Medicines Agency) in the EU and the FDA (Food and Drug Administration) in the USA.

The first guideline was issued on 10 March 1995 (and later revised on 20 February 1997), under the title “510(k) Information Needed for Hydroxyapatite Coated Orthopedic Implants” by the U.S. Department of Health and Human Services, FDA, Center for Devices and Radiological Health (CDRH)]. This document concisely outlines ten fundamental aspects related to hydroxyapatite coatings. In brief, particle size and distribution, pore volume and porosity, coating thickness and tolerance measured by scanning electron microscopy, chemical analysis of hydroxyapatite before and after coating (including Ca/P ratios and elemental analysis), coating adhesion strength, solubility products and dissolution rate of hydroxyapatite before and after processing, X-ray diffraction patterns, and infrared spectra pre- and post-coating. The tested coating must replicate as closely as possible the final product intended for the market distribution, including processing, all steps of cleaning, packaging and sterilization.

On 2 February 2000, the FDA issued a new industry guidance titled “Guidance for Industry on the Testing of Metallic Plasma Sprayed Coatings on Orthopedic Implants to Support Reconsideration of Postmarket Surveillance Requirements”, which focuses on metallic coatings and also references the relevant ASTM standards.

The most recent update from the FDA is represented by the draft guidance titled “Characterization of Metallic Coatings and/or Calcium Phosphate Coatings on Orthopedic Devices”. This document consolidates the two previous guidelines. Furthermore, unlike the 20 February 1997 guidance, it addresses calcium phosphate coatings more generally—not limited to hydroxyapatite—and provides an updated framework for their characterization and evaluation.

With regard to the International Organization for Standardization (ISO), the relevant standard is ISO 13779, issued in 2018 and divided into four parts, with the first version dating back to 2008 [212,213,214].

In relation to industrial scalability, the main characteristics considered by both the FDA guidelines and ISO 13779 include crystallinity, morphology, surface roughness and mechanical properties; primarily the adhesion of the coating to the substrate.

The crystallinity of coatings influences their solubility in physiological environment. Usually, the more crystalline the coating, the less soluble the CaP is in solution [215]. Soluble CaP coatings can promote the biomineralization process; however, it has been observed that rapid dissolution may negatively affect the long-term reliability of the implant, as well as lead to the premature release of biologically active substances or drugs [107]. Crystallinity can be controlled through post-deposition thermal treatment. However, if hydroxyapatite is functionalized with thermolabile molecules, sintering treatments cannot be applied. Moreover, depending on the sintering temperature, various phases may form in addition to calcium phosphate phases [216]. The international standard ISO 13779-2 recommends a crystallinity degree greater than 45% for calcium phosphate coatings used in bone implants. To maintain a low level of cytotoxicity, the amount of secondary phases (e.g., CaO) in calcium phosphate coatings should be less than 5% by weight. Methods for determining the crystallinity and the Ca/P ratio of calcium phosphate coatings and the content of secondary phases are thoroughly described in ISO 13779-3, as well as in ASTM F2024 Standard Practice for X-ray Diffraction Determination of Phase Content of Plasma-Sprayed Hydroxyapatite Coatings. Regarding coating dissolution, reference is made to ASTM F1926 Standard Test Method for Dissolution Testing of Calcium Phosphate Granules, Fabricated Forms, and Coatings [217,218].

The surface morphology and roughness and the thickness of CaP coatings influence the adhesion, growth, proliferation, and differentiation of both bone and bacterial cells. By adjusting deposition parameters and experimental conditions, the surface morphology of the coatings can be modified and optimized. Typically, CaP coatings with a surface roughness ranging between 0.5 and 1.5 μm are preferred, as they promote the cellular activity of osteoblasts or mesenchymal stem cells [219]. The porosity significantly impacts the bioactive behavior of bone implants in physiological environments. Pores larger than 100 μm (macroporosity) support tissue ingrowth through the coating and enhance the connectivity of newly formed bone cells. However, such large pores also considerably reduce the mechanical properties (e.g., compressive strength, shear resistance, and adhesion) [220]. The surface wettability is another important property that influences cell adhesion to the implant. Hydrophilic coatings tend to result in greater cell adhesion compared to hydrophobic ones [220]. Calcium phosphates are ionic solids, thereby promoting water spreading on the material surface. The ASTM F1854-15 Standard Test Method for Stereological Evaluation of Porous Coatings on Medical Implants (withdrawn in 2024) covered stereological methods for characterizing coating thickness, void content, and mean intercept length of various porous coatings adhering to nonporous substrates.

The adhesion of CaP coatings on the prosthesis surface is arguably the most critical requirement in the biomedical market. The performance of these coatings is assessed through tensile adhesion measurements, in accordance with international standard ISO 13779-4 or ASTM F1147 “Tension Testing of Calcium Phosphate and Metallic Coatings.” In Europe, the commercialization of orthopedic implants by industry manufacturers requires adhesion values greater than 15 MPa. In contrast, the FDA sets a stricter requirement for the U.S. market, mandating adhesion values exceeding 22 MPa [221].

Orthopedic implants are considered implantable devices that come into contact with tissues/bone for a long-term duration. Therefore, the following endpoints should be addressed in the biocompatibility evaluation: cytotoxicity; sensitization; irritation or intracutaneous reactivity; acute systemic toxicity; material-mediated pyrogenicity; subchronic toxicity (sub-acute toxicity); genotoxicity; implantation; chronic toxicity; and carcinogenicity. The relevant standard is ISO 10993-1 Biological evaluation of medical devices—Part 1: Evaluation and testing within a risk management process. Finally, after the validation of chemical–physical, mechanical, and biological characteristics has been completed, the in vivo evaluations are carried out, starting with non-clinical animal studies followed by clinical performance testing.

## 7. Discussion

In this literature review, the possibility to fabricate calcium phosphate-based coatings onto metallic implant and prosthesis surfaces was explored. In particular, the application of CaP-based coatings can improve the performance of medical devices in orthopedic and dentistry fields. Different calcium phosphate can be used to tuning the properties of calcium phosphate coatings, in term of crystallinity, solubility, trace ions element [7,8]. Regarding the possible application in orthopedic fields, different properties and features can be obtained coupling the CaP coatings with specific bioactive compounds and agents to increase the pro-osseointegration, antibacterial and antitumoral properties.

However, the research on the application of these innovative functionalized calcium phosphate coatings presents some limitations and future perspective. First, despite the presence of various calcium phosphates, hydroxyapatite remains the material mainly used for orthopedic applications. However, the different dissolution of specific CaP in contact with physiological environment can modulate the local ionic and bioactive molecules release [34,222]. Furthermore, TCPs and other CaP materials-based coatings have shown increases in mesenchymal stem cell viability, proliferation and osteogenic differentiation [223,224].

Another aspect that needs future study and focus regards the adhesion force of these coatings to the substrate surface. In fact, the adhesion force represents one of the most critical requirements for the application of these coatings in clinical trials and industrial scalability. As reported previously, the adhesion force needs to be higher than 15 MPa in Europe and 22 MPa for the FDA in the U.S market.

Finally, for the loading of bioactive components and agents for antibacterial and antitumoral applications, the correct release needs to be investigated to avoid the insurgence of antibiotic- and drug-resistance phenomena. In fact, often only the release into an in vitro environment for several days is explored to assess the presence of bioactive compounds.

## Figures and Tables

**Table 1:** Principal members of the calcium phosphate family relevant to orthopedic applications, classified according to their atomic Ca/P ratio, pH stability range in aqueous solutions at 25 °C, and density.

Ca/P Molar Ratio	Name	Formula	pH Stability Range	Density (g/cm^3^)	REF
1.0	**DCPA** (dicalcium phosphate anhydrous, monetite)	CaHPO_4_	2.0–5.5 (≥80 °C)	2.929	[14,15]
1.0	**DCPD** (dicalcium phosphate di-hydrate, brushite)	CaHPO_4_∙2H_2_O	2.0–6.0	2.319	[14,15,16]
1.33	**OCP** (octacalcium phosphate)	Ca_8_(HPO_4_)_2_(PO_4_)_4_∙5H_2_O	5.5–7.0	2.673	[15,16]
1.5	**α-TCP** (α-tricalcium phosphate)	α-Ca_3_(PO_4_)_2_	-	2.814	[15,16,17]
1.5	**β-TCP** (β-tricalcium phosphate)	β-Ca_3_(PO_4_)_2_	-	3.067	[15,16,18]
1.2–2.2	**ACP** (amorphous calcium phosphate)	Ca_x_H_y_(PO_4_)∙*n*H_2_O, *n* = 3–4.5, 15–20% H_2_O	5–12	-	[13,15]
1.5–1.67	**CDHA** (calcium deficient hydroxyapatite)	Ca_10−x_(HPO_4_)_x_ (PO_4_)_6−x_(OH)_2−x_ (0 < x < 2)	6.5–9.5	-	[15,19]
1.67	**HA** (Hydroxyapatite)	Ca_10_(PO_4_)_6_(OH)_2_	9.5–12	3.155	[10,13,15,16]
2.0	**TTCP** (tetracalcium phosphate)	Ca_4_(PO_4_)_2_O	-	3.056	[20,21]

**Table 2:** Properties in terms of thickness, morphology, topography, composition, adhesion strength and bioactivity of physical and chemical deposition techniques.

	Techniques Applied	Coating Properties				Ref
	Mechanism Source	Thickness Morphology and Topography	Ca/P ratio Crystallinity	Adhesion	Biocompatibility (in vitro)	
**Physical Deposition Techniques**						
PS	Molten powder spraying Thermal (T < 16,000 °C)	30–200 μm Molten microdroplets, partially fused spheroidal particles, possible pores or cracks	Alteration of stoichiometry (1.67) due to partial decomposition Variable—HA may undergo decomposition; improved with intermediate TiO_2_ layer	<35 MPa;	Good: promotes osteoblast adhesion, proliferation, and differentiation; positive in vitro tests (ALP, metabolism)	[1,10,15,73,78]
MS	Atomic sputtering with magnetic field Plasma	Between 40–50 nm and 3.5 µm Dense, amorphous, and pore-free film	Variable from 1.6 to 2.6, often higher than 1.67 Amorphous, dense pore-free coatings or highly oriented HA films	Generally 30 MPa; up to 60–80 MPa	Good: no cytotoxicity, good adhesion, proliferation, and viability of osteoblasts. Mineralization observed after 21 days in SBF. Differentiation of MSCs into osteoblast-like cells	[15,79,80,81,82]
PLD	Target ablation High-energy pulsed laser	50 nm—several µm -Excimer laser: → columnar structures -Nd:YAG laser → granular and robust morphology	~1.67 (ideal) at 0.1 Torr O_2_, can vary between 1.77 and 2.01 depending on pressure changes Amorphous at room temperature, can be improved through post-deposition thermal treatments	Very high: >58 MPa	Excellent: Stimulates osteoblast proliferation, promotes osseointegration, controlled in vitro dissolution	[15,34,80,83,84,85]
MAPLE	Thermally induced mechanical evaporation Pulsed laser	50–400 nm Particulate or slightly porous; surface roughness: 100–200 nm	~1.67 Typically amorphous or nanocrystalline		Excellent: Normal cell adhesion and morphology (e.g., SaOs2, MG63 cells), promoted osteodifferentiation (e.g., osteocalcin, type I collagen)	[75,86,87,88]
PED	Target ablation High-energy electron beam	300–800 nm Globular aggregates ranging from 20 to 80 nm; Roughness (Ra): 100–200 nm	Modifiable to values close to 1.67 Amorphous		Excellent: Supports cell proliferation, no cytotoxicity	[74,89,90]
IBAD	Physical deposition + ion beam Ion beam	Typically 2–4 µm -Dense film, free of visible pores or cracks; Maintains the roughness of the original substrate	Modifiable during deposition Amorphous	Very high, due to the intermixed interface and ion bombardment action	Excellent: No cytotoxicity, good cell adhesion and osteoblastic proliferation, effective mineralization in simulated body fluid (SBF)	[80,91,92]
IJD	Ionized jet from electric arc Plasma	300–700 nm Grains ~80 nm, aggregated into clusters up to 1.5 µm; Surface roughness: Hundreds of nanometers	Close to 1.67, faithfully maintained even with functional ions (e.g., Mg^2+^, Na^+^) present in biogenic apatites Amorphous		Excellent: promotes cell adhesion and proliferation, guides osteogenic differentiation of mesenchymal stem cells, demonstrated superior bioactivity compared to synthetic HA	[8,62,93]
**Chemical Deposition Techniques**						
Sol-gel	Chemical synthesis (sol-to-gel transition) + deposition (dip/spin/spray) Solution-based chemistry + thermal treatment	<1 µm Crystal shapes: platelets to circular petal-like clusters. Tunable roughness	Approximately 1.67 Nanocrystalline structure	>30 MPa	Good: supports osteointegration, May dissolve faster in SBF due to high porosity. Strategies to enhance stability: co-deposition with TiO_2_/ZrO_2_ or use of biodegradable polymers	[80,94,95]
BD	Chemical precipitation on pre-functionalized substrate (with –OH, –COOH groups or CaP seeds) Simulated Body Fluid (SBF)	From a few hundred nm to several µm (<30 µm) Flake-like or flower-like, porous. Surface Roughness: 100–300 nm	Tends toward 1.67 (HA), but may vary depending on the solution Initially low crystallinity, increases with prolonged immersion or post-deposition treatments	Weak if not pre-functionalized	High: supports osteoblast adhesion and proliferation. Ability to incorporate bioactive ions (Mg^2+^, Sr^2+^, Mn^2+^) and biomolecules (e.g., BMP-2, osteocalcin, fibronectin)	[63,96,97,98]
EPD and ECD	Electric-field-driven particle migration (EPD)/Electrochemical in situ growth (EC) Colloidal suspension (EPD)/Ion-containing electrolyte solution (EC)	50 nm–1 mm Depends on particle shape (e.g., spherical = fewer cracks); gradient structures achievable	Controllable and close to stoichiometric HA (1.67) Depends on post-deposition sintering (high temperatures required)	50–60 MPa	Excellent. Non-cytotoxic; supports osteoblast adhesion and growth in vitro. Supports cell adhesion and proliferation; enhanced bioactivity with additives like TiO_2_ or CNTs	[11,99,100,101]

**Table 3:** Example of different coating applications to improve osteoblast cell proliferation and reduce the resorption activity of osteoclast cells.

Calcium Phosphate Used	Deposition Technique	Bioactive Compounds Used	Application and Combination with CaP Coating	Biological Behavior	Ref
HA	Plasma Spray	Curcumin and Vitamin C	Drop casting	Increasing the osteoblast cell viability Reduction in osteoclast cell differentiation and osteosarcoma growth Antibacterial effect against *S. aureus*	[172]
HA	Matrix Assisted Pulsed Laser Evaporation	Alendronate	During MAPLE deposition	Increasing in the osteoblast-like cells proliferation and differentiation Inhibition of osteoclasts proliferation	[88]
HA	Electrophoretic deposition	Graphene oxide	During electrophoretic deposition	Increasing osteogenic differentiation Immune modulation	[174]
HA	Electrochemical deposition	Strontium, graphene oxide and linezolid	During electrochemical deposition	Osteoblast proliferation and differentiation	[179]
OCP	Matrix Assisted Pulsed Laser Evaporation	Alendronate	During MAPLE deposition	Reducing osteoclasts differentiation and proliferation Promotion of osteoblasts differentiation	[177]
HA	Electrochemical deposition	Phosphorylated osteogenic growth peptide	Grafting via chelation	Increasing osteogenic differentiation and migration	[175]
HA	Matrix Assisted Pulsed Laser Evaporation	Strontium and Zoledronate	During MAPLE deposition	Reduction of osteoclast proliferation and activity	[176]
HA	Plasma Spray	Zoledronic acid	Absorption	Reduction of osteoclast resorption activity	[178]

**Table 4:** Example of different loading of calcium phosphate-based coating to improve antibacterial properties.

Calcium Phosphate Used	Deposition Technique	Bioactive Compounds Used	Application and Combination with CaP Coating	Biological Behavior	Ref
HA	Electrochemical deposition	Simvastatin	During electrochemical deposition	Reduction in *S. aureus* biofilm formation	[156]
HA	Electrochemical deposition	Silver, BMP-2, chitosan	Electrostatic attraction and Absorption	Increasing antibacterial properties of *S. epidermidis* and *E. coli* Good osteoinductivity	[188]
HA	Hydrothermal method	Ampicillin and Vancomycin	Absorption	Inhibition against *E. coli* and *S. aureus* Increasing osteoblast-like cells growth	[188]
HA	Electrophoretic deposition	Vancomycin	During electrophoretic deposition	Increasing osteoblast cells viability Inhibition in *S. aureus* growth	[189]
HA	Plasma Spray	Vancomycin	Vacuum impregnation	Reduction in *S. aureus* growth Increasing osteoblast-like cells viability	[190,191]
HA	Sol-Gel	Ciprofloxacin	During sol-gel deposition	Reduction in *E. coli* growth Increasing osteoblast-like cells viability	[192]
HA	Electrophoretic deposition	Ciprofloxacin	Absorption	Antibacterial properties against *E. coli* and *S. aureus*	[193]

**Table 5:** Example of coating applications to improve the cytotoxicity against cancer cells.

Calcium Phosphate Used	Deposition Technique	Bioactive Compounds Used	Application and Combination with CaP Coating	Biological Behavior	Ref
HA	Micro Arc Oxidation	Paclitaxel	Absorption	Toxic effect against human cervical carcinoma cells	[201]
HA	Plasma Spray	Curcumin and Epigallocatechin	Drop-casting method	Cytotoxic effect against osteosarcoma cells	[205]
HA	Plasma Spray	Curcumin and Vitamin D3	Absorption	Cytotoxic effect against osteosarcoma cells	[206]
HA	Matrix Assisted Pulsed Laser Evaporation	Carboplatin and Quercetin	During MAPLE deposition	Cytotoxic effect against osteosarcoma cells	[207]
HA	Micro Arc Oxidation	Selenium	During micro arc oxidation deposition	Cytotoxic effect against cancer cells	[208]

## Data Availability

No new data were created.
